# Efficient Blockade of Akt signaling is a determinant factor to overcome resistance to Matuzumab

**DOI:** 10.1186/1476-4598-10-151

**Published:** 2011-12-20

**Authors:** Debora D Meira, Vitor H Almeida, Jânio S Mororó, Mauricio S Caetano, Isabel P Nóbrega, Delano Batista, Cinthya Sternberg, Carlos G Ferreira

**Affiliations:** 1 Coordination of Clinical Research, Instituto Nacional de Câncer (INCA), Rio de Janeiro, Brazil; 2 Departamento de Farmácia, Escola Superior de Ciências da Santa Casa de Misericórdia de Vitória (EMESCAM), Espírito Santo, Brazil

**Keywords:** Matuzumab, PI3K/Akt pathway, EGFR, gynecological cancer, cervical cancer, Cetuximab

## Abstract

**Background:**

Clinical studies have shown antineoplastic effectiveness of monoclonal antibodies (MAbs) against EGFR for different indications. Several MAbs directed to EGFR were developed recently, such as matuzumab, but there is still lack of information on preclinical data on its combination with chemo-radiation. Thus, the present study intended to examine the molecular pathways triggered by matuzumab alone or associated to chemo-radiotherapy in gynecological cell lines and its impact on cell growth and signaling.

**Results:**

Combination of matuzumab with radiation and cisplatin did not enhance its cytostatic effects on A431, Caski and C33A cells (high, intermediate and low EGFR expression, respectively) in clonogenic assays, when compared to controls. The lack of effect was mediated by persistent signaling through EGFR due to its impaired degradation. In spite of the fact that matuzumab inhibited phosphorylation of EGFR, it had no effect upon cell viability. To analyze which downstream molecules would be involved in the EGFR signaling in the presence of matuzumab, we have tested it in combination with either PD98059 (MAPK inhibitor), or LY294002 (PI3K inhibitor). Matuzumab exhibited a synergic effect with LY294002, leading to a reduction of Akt phosphorylation that was followed by a decrease in A431 and Caski cells survival. The combination of PD98059 and matuzumab did not show the same effect suggesting that PI3K is an important effector of EGFR signaling in matuzumab-treated cells. Nonetheless, matuzumab induced ADCC in Caski cells, but not in the C33A cell line, suggesting that its potential therapeutic effects *in vitro *are indeed dependent on EGFR expression.

**Conclusions:**

Matuzumab combined with chemoradiation did not induce cytotoxic effects on gynecological cancer cell lines *in vitro, *most likely due to impaired EGFR degradation. However, a combination of matuzumab and PI3K inhibitor synergistically inhibited pAkt and cell survival, suggesting that the use of PI3K/Akt inhibitors could overcome intrinsic resistance to matuzumab *in vitro. *Altogether, data presented here can pave the way to a rational design of clinical strategies in patients with resistant profile to anti-EGFR inhibitors based on combination therapy.

## Introduction

Epidermal growth factor receptor (EGFR), a 170-kDa transmembrane glycoprotein, belongs to the ErbB/HER family of receptors which includes HER2 (ErbB2/neu), HER3 (ErbB3) and HER4 (ErbB4). Ligand binding leads to the formation of homo or heterodimers between members of the family, facilitating receptor autophosphorylation. Phosphorylated receptors subsequently activate signaling pathways that regulate cell proliferation, survival and transformation [1,2]. EGFR inhibition by anti-EGFR monoclonal antibodies (MAbs) or tyrosine kinase inhibitors (TKIs) represents a particularly successful molecular targeted therapy for tumors such as Non-Small Cell Lung Cancer and Colorectal Cancer.

Anti-EGFR MAbs bind EGFR with higher affinity than the original ligands, preventing receptor activation. Moreover, they induce EGFR internalization and degradation, with consequent cell cycle arrest, inhibition of proliferation and angiogenesis, and promotion of *in vitro *and *in vivo *antibody-dependent cellular cytotoxicity (ADCC) [3]. Although exhibiting a plethora of antineoplastic mechanisms, numerous reports have described that several patients using EGFR inhibitors experience an initial clinical response followed by disease progression [4,5]. In spite of the benefits experienced by most patients bearing EGFR mutations, some of them will already present intrinsic resistance to EGFR-targeted therapy at diagnosis.

Recently, several studies have shed light upon the mechanisms of acquired resistance to anti-EGFR MAbs and TKIs, and among them, the most important are the incidence of *EGFR *mutations [6,7], altered mechanisms of internalization and down-regulation of EGFR [6-8], inability of MAbs to prevent the formation of ligand-induced heterodimers [4], *KRAS *mutations [9] and *PTEN *loss [4]. These mechanisms culminate in a sustained activation of major intracellular signaling pathways controlled by MAPK and Akt, leading to persistent cell survival [10]. Altogether, data suggest that altered signal transduction emerges as a major driving force in molecular target drug resistance and, therefore, one can expect that resistance could be overpowered by the combined use of specific inhibitors targeting such pathways in cancer cells.

Matuzumab, a humanized IgG1 derived from the murine precursor EMD 55900 (MAb 425), binds to EGFR with high affinity [11] and, to the best of our knowledge, data on the combination of matuzumab plus chemoradiation are lacking. In this study, we sought to analyze the effects of matuzumab, either alone or combined with cisplatin and/or radiotherapy, on gynecological epidermoid carcinoma cell lines expressing distinct EGFR protein levels [12]. Here we show that matuzumab combined with chemoradiation did not enhance cytotoxic effects on gynecological cancer cells lines. In spite of inhibiting autophosphorylation, matuzumab was not able to induce EGFR down-regulation and persistent activation of downstream signaling pathways was observed. Accordingly, we analyzed the activation of downstream targets of EGFR to determine the partners involved in the signaling pathway elicited by EGF in the matuzumab-treated cells. In this setting, PI3K/Akt pathway inhibition, unlikely MAPK inhibition, sensitizes gynecological cancer cells to matuzumab treatment *in vitro*. These results reinforce the paradigm that several signal transduction pathways control tumor growth and contribute to resistance. Therefore, future therapeutic approaches are likely to involve the combination of different antineoplastic targeted agents.

## Materials and methods

### Cell lines

A431 human cell line (vulvar carcinoma) was kindly provided by Dr. Giuseppe Giaccone (University Hospital Vrije Universiteit, The Netherlands). Caski and C33A human cells (cervical carcinoma) were provided by Dr. Luisa L. Villa (Ludwig Institute for Cancer Research, SP, Brazil).

### Chemicals

Matuzumab and cetuximab were generously provided by Merck KGaA (Darmstadt, Germany). PD98059, LY294002 and MG132 were purchased from Calbiochem (Nottingham, UK).

### Analysis of EGFR cell surface expression by flow cytometry

As previously described [13], cells were incubated either with a murine anti-EGFR Mab (0.1 μg/uL, BD Pharmingen, San Diego, CA) or matuzumab (0.1 μg/uL) for 1 h on ice. After washing, secondary antibodies (Caltag Laboratories, Burlingame, CA) were added and samples were analyzed on a FACScalibur using CELLQuest software (Becton Dickinson, San Jose, CA).

### MTT and clonogenic assays (CA)

For the MTT (3-(4,5-dimethythiazol-2-yl)-2,5-diphenyltetrazolium bromide) assay, Caski and C33A cells were incubated with matuzumab at different concentrations, or matuzumab in the presence/absence of 25 μM of PD98059, a MEK1/2 inhibitor [14]. To compare matuzumab with cetuximab effects, A431, Caski and C33A cells were incubated with 100 μg/mL of either antibody. After 72 h, cells were incubated with a solution of MTT (Sigma, St. Louis-MO), processed as previously described [15]. Cell viability was expressed as a percentage of controls (%CT).

For the combination experiments in CA, A431, Caski and C33A cells were incubated with matuzumab (100 μg/mL) and LY294002 (10 μM) during the whole colony formation assay. Alternatively, matuzumab (100 μg/mL) and cisplatin (1.0, 0.5 and 0.15 μM for A431, Caski and C33A cells, respectively) were added and cells were irradiated 6 h later (2.0, 1.5 and 0.3 Gy for A431, Caski and C33A cells, respectively) with a ^60^Co-THERATRON-780C irradiator (Theratronics, Canada), and maintained at 37°C for 72 h. Each cell line was irradiated at different intensities and also treated with different doses of cisplatin according to the specific sensitivities of each cell line, as previously described [12]. For experiments comparing matuzumab to cetuximab, cells were incubated with 100 μg/mL of either antibody for 72 h. Cells were then kept in fresh medium for 10 days and the number of colony-forming units stained with crystal violet was expressed as the surviving fraction (SF), processed as previously described [13].

### Cell cycle analysis

Cells were incubated in the presence of matuzumab (100 μg/mL), as previously described [10]. After 24 h, cell cycle phase distribution was analyzed by flow cytometry using propidium iodide (PI) staining and the resulting DNA content was analyzed on a Becton Dickinson FACScalibur using ModFitLT V2.0 software (Becton Dickinson, San Jose, CA).

### Western blotting (WB) analysis

Cells were maintained in culture medium containing 10% FBS v/v and prior to MAb treatments and were starved for 18 h in culture medium supplemented with 1% FBS v/v. Low serum concentration was used to reduce signaling elicited by growth factors in the serum, while ensuring survival of cells [16]. Prior to growth factor stimulation, cells were incubated for a period of 4 h in serum-free medium in the presence of matuzumab (100 μg/mL) alone or followed by a 15-minutes incubation with EGF (10 ng/mL) as previously described [13]. For combination experiments, cells were treated as described above, plus 1 h of incubation with either PD98059 (25 μM) or LY294002 (10 μM), alone or combined with matuzumab before the incubation with EGF. For EGFR degradation analysis, as described by others [17], A431 and Caski cells were incubated with either matuzumab or cetuximab (100 μg/mL, each) for 24 h in serum-free culture medium and when indicated in the figure, 15 μM of MG 132 (a proteasome inhibitor) was added for the last 6 h in combination with either MAb. Primary antibodies against total and phosphorylated EGFR, HER2, Akt and MAPK (all from Cell Signaling Technology, Beverly, MA, USA) were used. Immunoblots were developed using the enhanced chemoluminescence (ECL) reagent (GE Health Care, SP, Brazil) and bands were quantified with Labworks, version 4.6 (Bio-Rad, USA).

### Annexin V staining

Cells were incubated in the presence of matuzumab (100 μg/mL) or/and LY 294002 (10 μM). After 72 h, apoptosis was analyzed by flow cytometry using annexin V staining (BD Biosciences) on a Becton Dickinson FACScalibur (Becton Dickinson, San Jose, CA).

### *In vitro *ADCC assay

ADCC assay was performed with the kit CytoTox96^® ^Non-Radioactive Cytotoxicity Assay (Promega, Madison, WI). Cells were incubated alone or in the presence of 4 μg/mL of matuzumab for 4 h and exposed to peripheral blood mononuclear cells (PBMC, effector cells) at effector/target ratio (E/T) of 20:1 for 4 h and specific cytolysis (ADCC) was measured as previously described [13].

### Statistical analysis

All experiments were performed in triplicates and the values represent an average of at least 3 independent experiments. Statistical analyses were performed using GraphPad Prism 3.0 (GraphPad Software Incorporated, San Diego, CA, USA). Quantitative experiments were analyzed by Student's *t *test. One-Way analysis of variance (ANOVA) with Tukey's post test was used to analyze the combination of matuzumab, cisplatin and RxT versus double or individual treatments by CA. All *P *values resulted from the use of two-sided tests and were considered significant when < 0.05 or < 0.0001.

## Results

### A431, Caski and C33A cells differentially express EGFR

**Figure 1:**
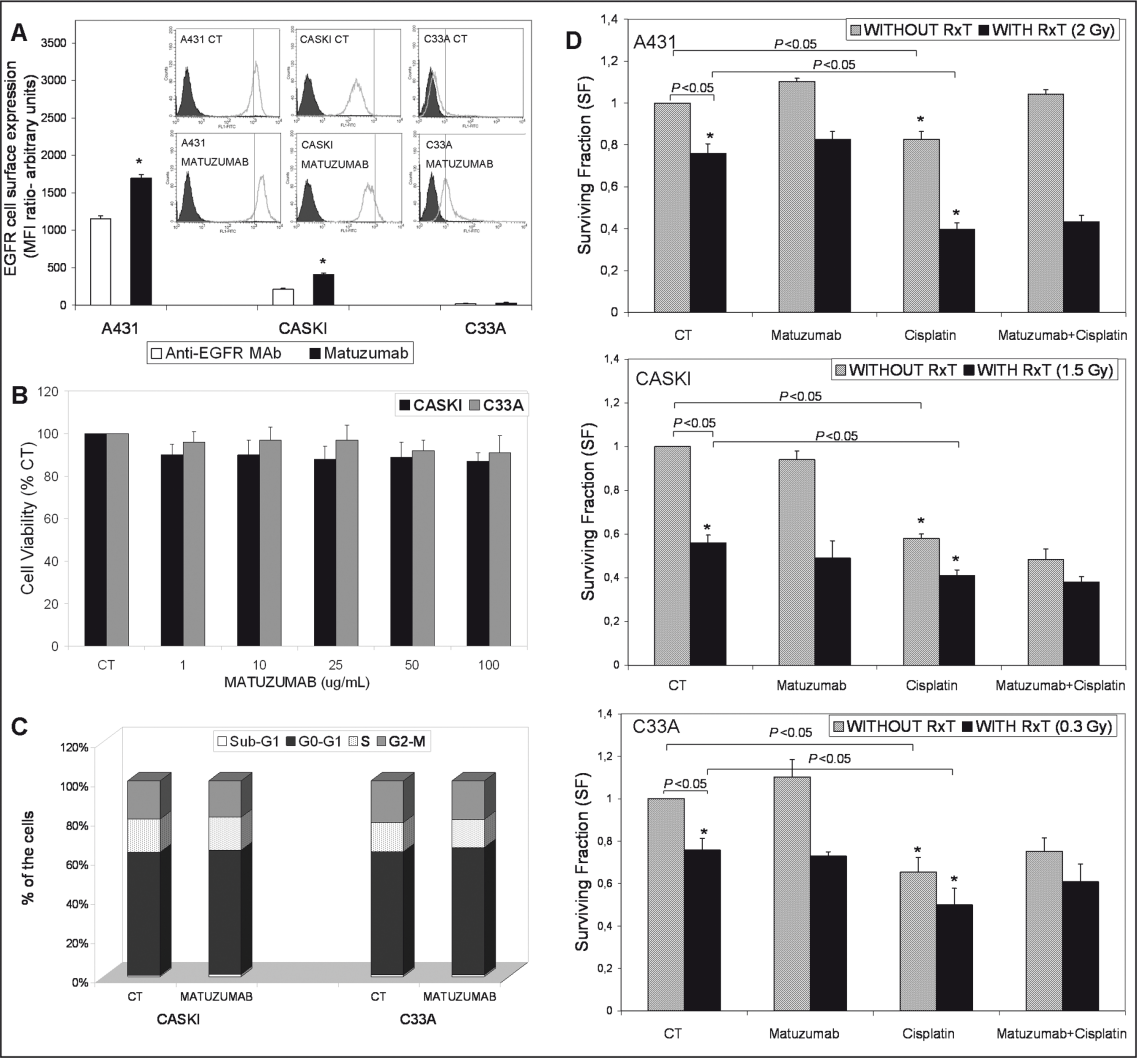
**Matuzumab fails to induce chemo/radio sensitization of A431, Caski and C33A gynecological cancer cell lines**. (A) EGFR expression detected by Matuzumab on FACS analysis of A431, Caski and C33A cells, Student's t test * *P *< 0.05, when compared to controls. (B) Effects of matuzumab treatment (100 μg/mL) in Caski and C33A cells by MTT assay and (C) on cell cycle phase distribution analysis by flow cytometry. (D) Effects of matuzumab alone (100 μg/mL) or combined with cisplatin (1.0, 0.5 and 0.15 μM for A431, Caski and C33A cells, respectively) and/or RxT on colony formation by clonogenic assay (CA). One-Way ANOVA analysis of variance with Tukey's post test * *P *< 0.05, when compared to control cells.

Previously, we have shown by Real Time PCR analysis that A431 cells exhibit abnormally high expression of EGFR, Caski cells express intermediate levels of EGFR mRNA, whereas C33A cells express the lowest levels of such molecule [12]. To further characterize the expression of EGFR in these cells, we have examined cell surface EGFR expression by FACS and observed that both a murine anti-EGFR MAb and matuzumab were able to detect elevated, intermediate and low levels of membrane-bound EGFR on A431, Caski and C33A cells, respectively (Figure 1A).

### Matuzumab does not inhibit cervical cancer cell proliferation

In a previous study, we have demonstrated that matuzumab was not able to inhibit A431 cells proliferation, nor it caused significant changes in cell cycle distribution [13]. In the present study, we also observed that matuzumab treatment did not decrease viability of cervical cancer (CC) Caski and C33A cells accessed by MTT assay, regardless of the concentration used (Figure 1B). Also, there was no effect upon cell population distribution among the cell cycle phases in Caski and C33A cells when compared to controls (Figure 1C).

### Matuzumab did not sensitize A431, Caski and C33A cells to chemo/radiotherapy

We evaluated whether the combination of matuzumab (100 μg/mL) and radiotherapy (RxT) and/or cisplatin could enhance the cytotoxic effects observed with the isolated treatments on the A431, Caski and C33A cells. Cisplatin and RxT either alone or combined decreased the survival of all cell lines tested (*P *> 0.05, Figure 1D). However, the combination of matuzumab with either RxT or cisplatin was not able to enhance the cytotoxic effects of the isolated treatments, and neither triple combination of matuzumab, RxT and cisplatin was able to enhance the cytotoxicity of combined treatment with cisplatin and RxT (Figure 1D).

### Matuzumab inhibits EGFR and HER2 phosphorylation

**Figure 2:**
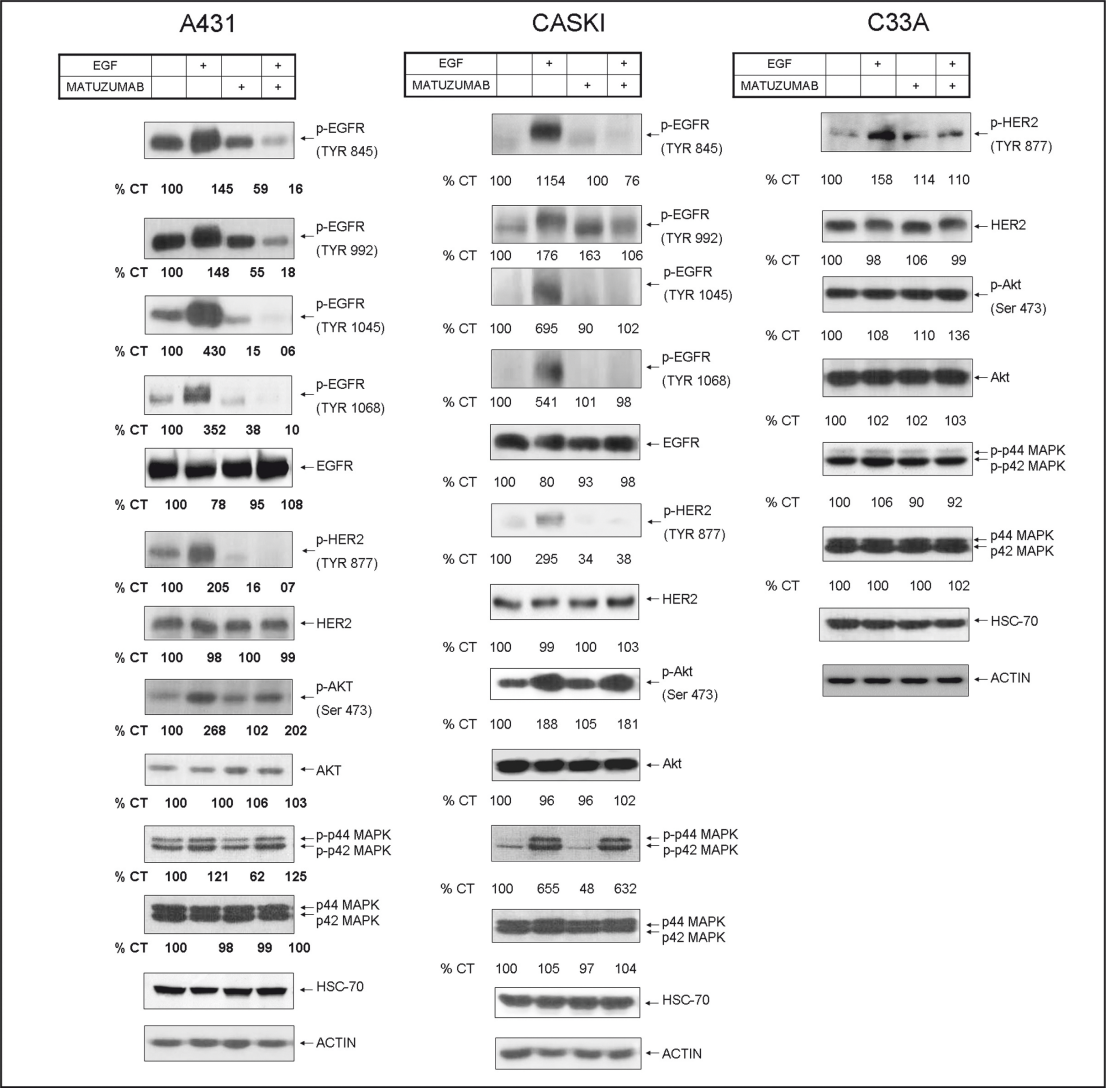
**Matuzumab inhibits EGFR and HER2 phosphorylation, but not Akt and MAPK phosphorylation elicited by EGF**. Effects of Matuzumab (100 μg/mL) on EGF-induced activation of EGFR (Tyr 845, 992, 1045 and 1068), HER-2/*neu*, Akt and ERK 1/2 on A431, Caski and C33A cells, detected by Western blotting.

As matuzumab did not exert any effects on cell proliferation of the gynecological cancer cell lines tested (Figure 1 and reference 13), we sought to analyze the phosphorylation state of EGFR receptor, as it ultimately dictates its activation status. EGFR phosphorylation was analyzed by WB in cells treated with matuzumab alone (100 μg/mL) or in the presence of EGF. Receptor phosphorylation was increased by EGF treatment in A431 and Caski cells, while matuzumab strongly inhibited it at least in 3 out of the four residues analyzed (Figure 2). Also, EGF induced a slight decrease in the total amount of EGFR in these cell lines, whereas matuzumab did not (Figure 2). EGFR can interact with another member of the ErbB family, HER2, an orphan receptor, to form heterodimers that are very potent in activating signal transduction pathways [1]. Following matuzumab treatment, there were no changes in total HER2 expression in A431, Caski and C33A cell lines, however, EGF-induced HER2 phosphorylation was inhibited by matuzumab in A431 and Caski cell lines (Figure 2). Interestingly, in C33A cells, that do express HER2 but not EGFR [12], matuzumab treatment induced a slight reduction of EGF-induced HER2 phosphorylation (Figure 2).

### Matuzumab fails to inhibit Akt and ERK 1/2 phosphorylation elicited by EGF

Matuzumab treatment did not affect the overall expression of Akt and MAPK in the gynecological cancer cell lines tested (Figure 2). Akt and ERK 1/2 phosphorylation was increased by EGF treatment in A431 and Caski cells, but not in C33A cells. There were no changes in the phosphorylation state of the above mentioned kinases when cells were treated with EGF in the presence of matuzumab (Figure 2). Altogether, these data suggest that persistent signaling through the Akt and MAPK pathways, even in the presence of matuzumab, lead to increased survival of Caski and C33A cells, corroborating the results obtained in the MTT assay and cell cycle analysis (Figure 1).

### Matuzumab does not induce EGFR down-regulation

**Figure 3:**
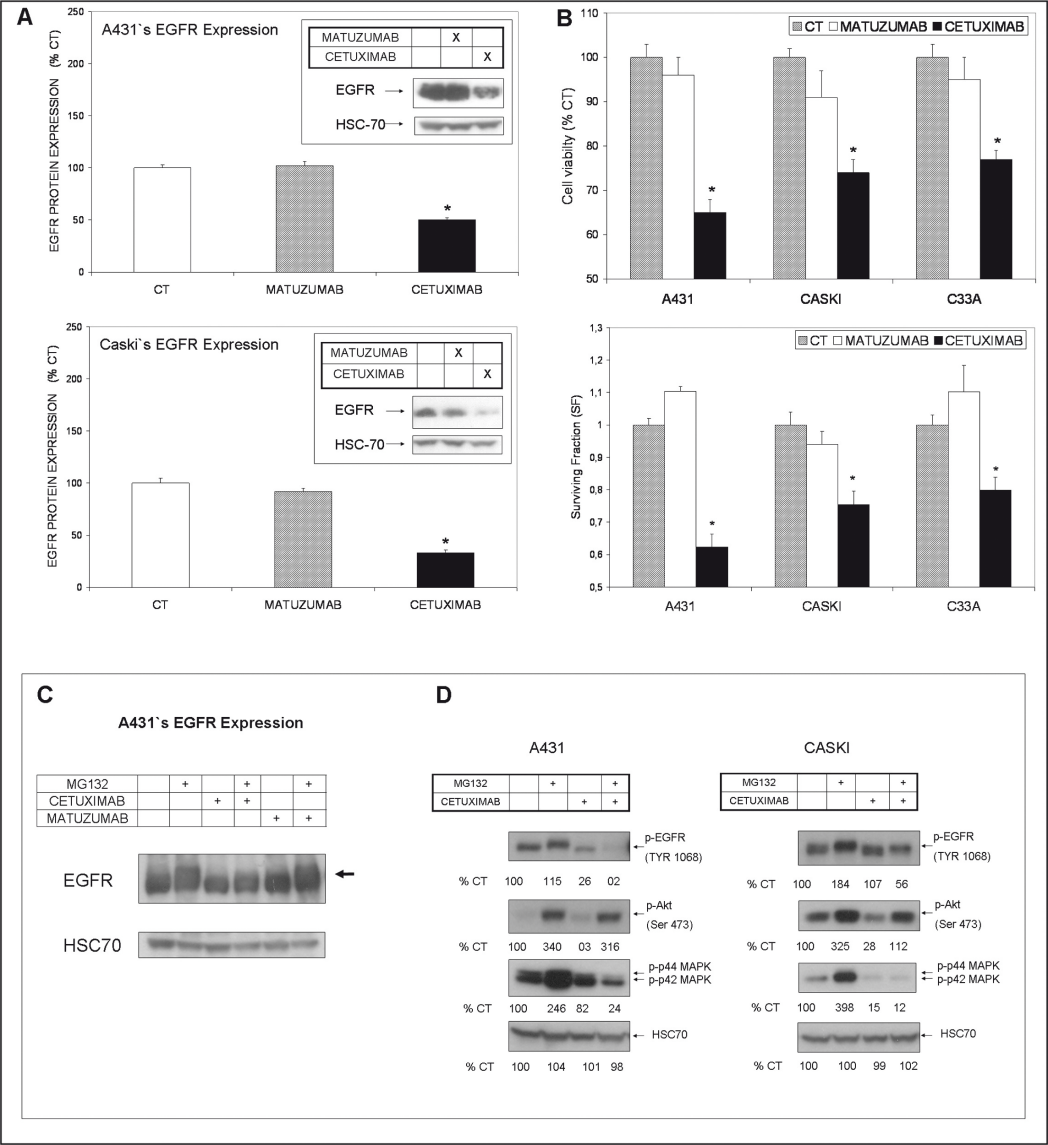
**Differences between matuzumab and cetuximab regarding the modulation of EGFR down-regulation, cell proliferation and survival**. (A) A431 and Caski cells were incubated with matuzumab or cetuximab (100 μg/mL, each) for 24 h in serum-free culture medium and cells were then lysed for Western blotting analysis of total EGFR. (B) Effects of matuzumab or cetuximab (100 μg/mL, each) on cell metabolic viability (MTT assay) and colony formation (clonogenic assay) of A431, Caski and C33A cells. Student's t test * P < 0.05, when compared to control cells. (C) Effects of MG132 (15 μM), a proteassomal inhibitor, in combination with cetuximab or matuzumab (100 μg/mL, each) on EGFR expression of A431 cells, detected by Western blotting. (D) Effects of MG132 (15 μM) in combination with cetuximab (100 μg/mL) on phosphorylation of EGFR (Tyr 1068), Akt and ERK1/2 on A431 and Caski cells, detected by Western blotting.

Endocytosis and receptor degradation induced by anti-EGFR MAbs culminate in the inactivation of growth factor receptors and suppression of downstream signaling pathways, reducing the proliferative/survival potential of cancer cells [3,17,18]. As the anti-EGFR MAb cetuximab efficiently induces EGFR degradation [8,17] and subsequent decrease cell survival [3,12], it was used as a positive control to investigate if matuzumab could induce EGFR down-regulation. A431 and Caski cells were treated with either matuzumab or cetuximab (100 μg/mL) for 24 h. C33A cells were not included in this experiment, since its EGFR expression is nearly undetectable by WB. As expected, 24 h-treatment with cetuximab induced a robust reduction of 50% and 70% in EGFR protein content in A431 and Caski cells, respectively (Figure 3A). As a proof of concept, we have treated A431 cells with MG132, a proteassomal inhibitor, and observed that EGFR accumulates both in its total (Figure 3C) and in its phosphorylated form (Figure 3D), and a shift in the EGFR band is observed, probably due to the increase in molecular weight caused by conjugation of ubiquitin molecules to the receptor (indicated by the arrow in Figure 3C). The same result was observed in Caski cells (data not shown). pEGFR accumulation induced an increase both in pERK and pAkt, implicating EGFR accumulation in the persistent activation of cell signaling pathways elicited by this receptor (Figure 3D), however cetuximab only inhibited pERK increase but not pAkt increase in the presence of proteassomal inhibitor in both cells. In contrast, treatment with matuzumab for 24 h failed to induce EGFR down-regulation in both cell lines (Figure 3A), demonstrating that this event is independent of the cell type analyzed (as stated previously, Caski is a cervical carcinoma and A431 is a vulvar carcinoma). Of note, the lack of EGFR down-regulation after 24 h of matuzumab treatment could explain the sustained cell proliferation and survival observed in the cell cycle analysis, MTT and CA assays (Figures 1 and 3B).

### Combination of matuzumab with PD98059, a MAPK inhibitor, induces antagonistic effects in A431, Caski and C33A cells

**Figure 4:**
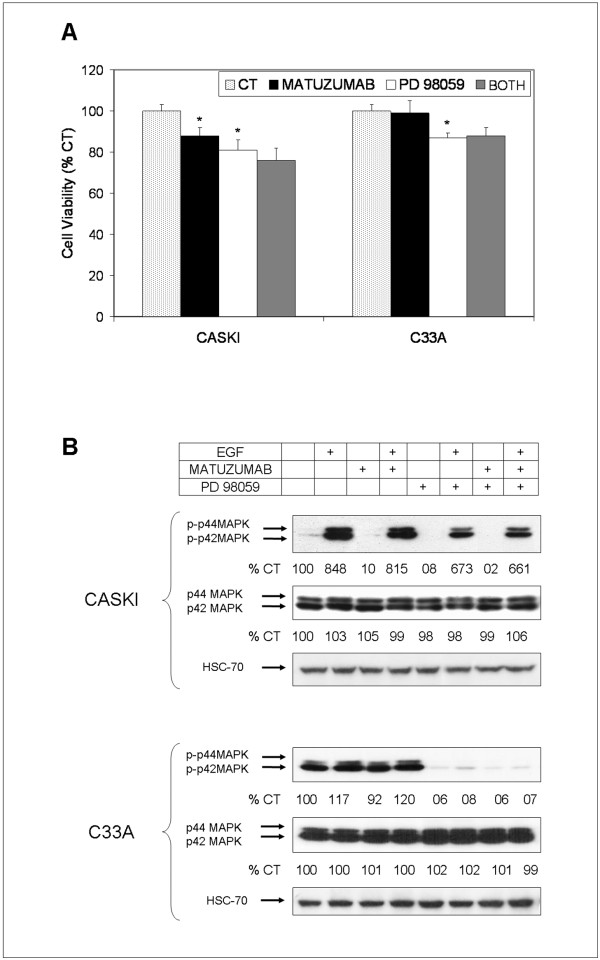
**Combination of matuzumab with PD98059, a MAPK inhibitor, induces antagonistic effects in A431, Caski and C33A cells**. (A) Effects of combined treatment of matuzumab (100 μg/mL) with a MAPK pathway inhibitor, PD98059 (25 μM), on cell viability by MTT assay and (B) Western blotting analysis of phosphorylation of p44/42 (ERK) 1/2 on Caski and C33A cells.

A major signaling route of EGFR is the mitogen-activated protein kinases (MAPK) pathway and its overactivation plays a critical role in tumor development and progression [14]. Since we observed that matuzumab could not reduce MAPK phosphorylation elicited by EGF (Figure 2), we speculated that combination of matuzumab and PD98059, a specific MEK1/2 inhibitor, could decrease cell viability over single-drug treatments. Although PD98059 treatment alone decreased cell viability and ERK 1/2 phosphorylation of Caski and C33A cells, isolated matuzumab did not (Figures 4A and 4B). Surprisingly, there was no significant statistical difference between isolated and combined treatments in Caski and C33A cell survival (Figure 4A), with no further decrease in ERK 1/2 phosphorylation status of combined over single drug exposure (Figure 4B). We have previously shown that matuzumab and PD98059 failed to cooperate in reducing the cell viability of A431 cells [13]. These results reinforce the idea that matuzumab effects upon phosphorylation of EGFR, but not EGFR degradation, are not modulating the persistent MAPK signaling. This might be due to the fact that EGFR phosphorylation is not completely abolished by matuzumab and since the receptor is not degraded by the MAb, matuzumab continues inducing cell signaling and sustaining cell proliferation.

### Blockade of Akt signaling is a determinant factor to overcome resistance to matuzumab

**Figure 5:**
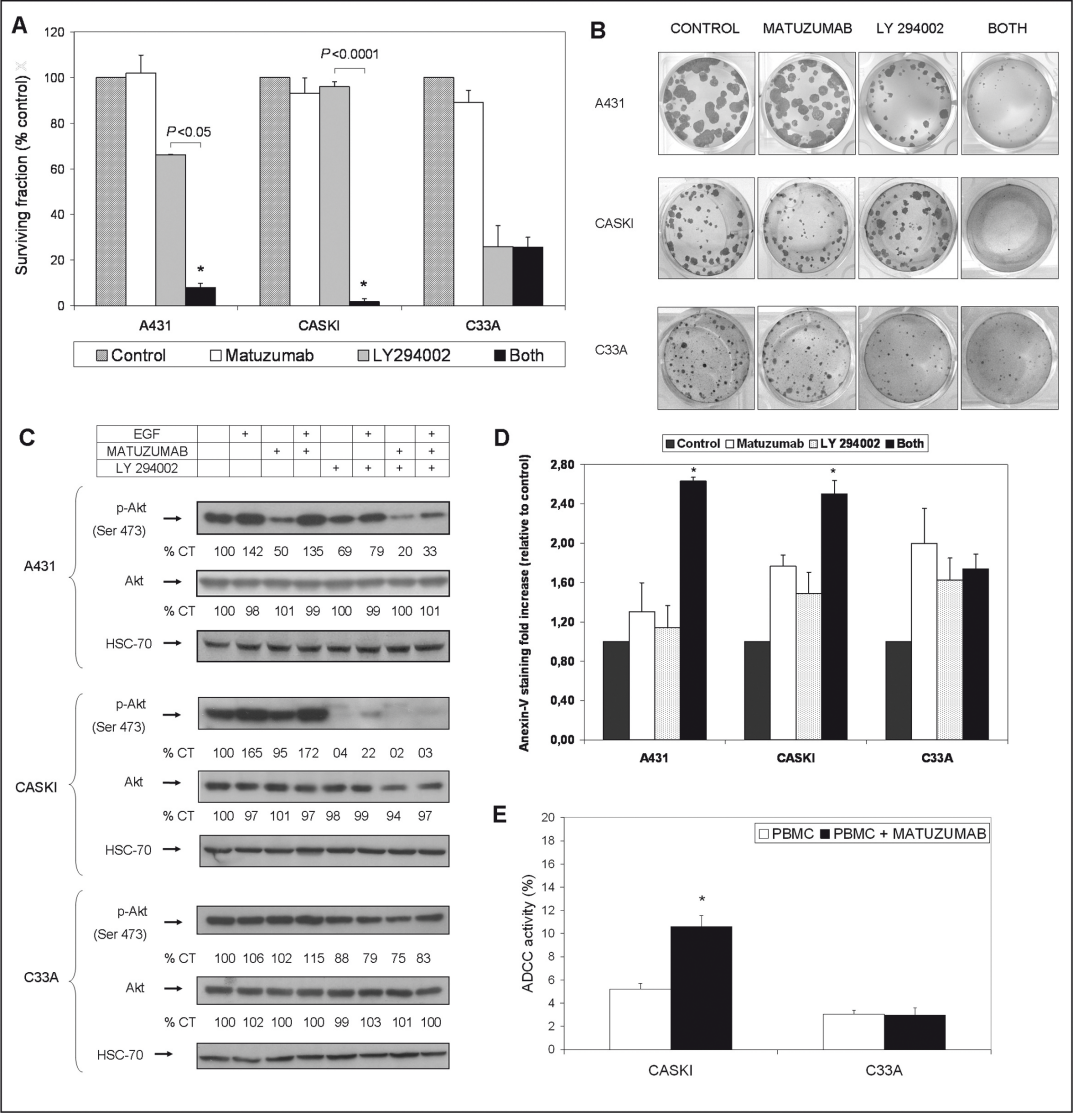
**Targeting PI3K could overcome resistance to matuzumab**. (A) Effects of combined treatment of matuzumab (100 μg/mL) with a PI3K inhibitor, LY294002 (10 μM), on the colony formation of A431, Caski and C33A cells by clonogenic assay (CA). (B) Representative pictures of colonies of A431, Caski and C33A cells after treatments with matuzumab (100 μg/mL) and/or LY294002 (10 μM) in CA and (C) WB analysis of total and phosphorylated Akt (Ser 473) on A431, Caski and C33A cells. (D) Effects of combined treatment of matuzumab and LY 294002 on the apoptosis of gynecological cancer cell lines analyzed by annexin V staining. (E) Cells were incubated alone or in the presence of 4 μg/mL of matuzumab for 4 h and exposed to peripheral blood mononuclear cells (PBMCs, effector cells) at effector/target ratio (E/T) of 20:1 for more 4 h and specific cytolysis (ADCC) was measured. Student's t test * *P *< 0.05, when compared to control cells.

Previous results of our group showed that when in combination to cetuximab, that triggered EGFR degradation, matuzumab induced further reduction in cell signaling and survival when compared to cetuximab alone [13]. These results implicate that matuzumab binding to EGFR induces distinct inhibitory effect to the ones induced by cetuximab. Additionally, several reports have described that the PI3K/Akt pathway remained active and was involved in the lack of sensitivity to EGFR inhibitors in different cell types [5,10,19]. Since diverse signal transduction pathways control tumor resistance to antineoplastic agents, we hypothesized that, unlikely the MAPK inhibitor PD98059, a PI3K-Akt pathway inhibitor could decrease cell survival in the presence of matuzumab. Based on this assumption, we investigated whether the use of LY294002, a phosphatidylinositol 3-kinase (PI3K) inhibitor, could overpower resistance to matuzumab *in vitro*. As predicted, combined treatments strongly reduced A431 and Caski cell survival (92% and 98% of inhibition, respectively) leading to a markedly reduction in number and size of A431 and Caski colonies (*P *< 0.05 and *P *< 0.0001, respectively) when compared to either treatments alone (Figures 5A and 5B). Additionally, the combination of LY294002 and matuzumab in A431 and Caski cells was accompanied by a markedly reduction of Akt phosphorylation, with no changes in total Akt protein expression (Figure 5C).

In contrast, we have demonstrated that the combination of cetuximab and PD153035 (a specific EGFR TKI) proved to be antagonistic in C33A cell line, with no reduction in proliferation and EGFR, HER2, AKT and MAPK phosphorylation status when compared to either drug alone [12]. Previously, we demonstrated that C33A cells do not rely on EGFR signaling to proliferate and that cetuximab has no effect upon EGFR, HER2, AKT and MAPK phosphorylation status, and even the combination of cetuximab and the EGFR-specific tyrosine kinase inhibitor PD153035, did not display enhanced toxicity when compared to either agent alone [12]. Here, we observed that there was no significant difference in the proliferation of C33A cells treated with LY294002 combined with matuzumab compared to LY294002 treatment (*P *= 0.9076; Figures 5A and 5B), neither there was a decrease in Akt phosphorylation elicited by EGF in cells exposed to the combined treatment (Figure 5C), when compared to LY294002. As PI3K-Akt pathway activation leads to cell survival [4], we evaluated whether the combination of matuzumab and LY294002 was able to induce apoptosis, which would explain the synergistic effect of these drugs observed in A431 and CASKI cell lines. One of the earliest features of apoptosis is the translocation of phosphatidylserine from the inner to the outer leaflet of the plasma membrane. Apoptosis was measured by annexin V staining, since annexin V binds to phosphatidylserine exposed on the cell surface and identifies cells at an earlier stage of apoptosis. In the A431 and CASKI cell lines, but not in C33A cells, there was an increased induction of apoptosis by combined treatment with matuzumab and LY 294002 compared to isolated treatments (Figure 5D). Altogether, these data corroborate the hypothesis that resistance to matuzumab in EGFR expressing cells, such as A431 and Caski, could be modulated by agents that disrupt the persistent downstream signaling pathways observed here. PI3K pathway-targeted therapies, which will ultimately lead to an efficient blockade of Akt activation, may become promising drugs to manage resistance to matuzumab in gynecological oncology clinics.

### Matuzumab induces ADCC in Caski cell line, but not in C33A cells

ADCC is an important *in vivo *mechanism of cell-mediated immunity whereby an effector cell of the immune system actively lyses a target cell that has been recognized by specific antibodies. It is one of the mechanisms through which anti-EGFR antibodies can act to limit and contain tumor growth. The ADCC phenomenon is dependent on the number of EGFR molecules per cell and how efficiently they are recognized by antibodies [9,12,20]. FACS analysis showed that matuzumab detected a larger amount of cell surface receptors than the anti-EGFR antibody in A431 and Caski cells (Figure 1A, *P *< 0.05). In C33A cells, matuzumab was able to detect a small amount of EGFR molecules per cell, but there was no substantial difference when compared to the control (Figure 1A). Accordingly, at Effector/Target (E/T) ratio of 20:1, matuzumab mediated lysis in 10.6% of Caski cells, but not in C33A cells (3.1%) (Figure 5E). Thus, in spite of the lack of effects upon EGFR signaling, ADCC induced by matuzumab is dependent on cell surface expression of EGFR and this event could account for its partial effectiveness in clinical trials so far [21-23]

## Discussion

In the last decades, research in cancer generated a major progress in the understanding of the molecular basis of cancer that, along with biotechnology advances, allowed the development of new antineoplastic targeted agents and a subsequent improvement in cancer treatment. Despite the progress, mechanisms of resistance to cancer therapy either inherited or acquired remain a hurdle, requiring new strategies to overcome it. The anti-EGFR MAb matuzumab was tested in early clinical trials in some tumor types, even though the preclinical data supporting its antitumor efficacy was scarce. The present report, to the best of our knowledge, is the first one to show that matuzumab does not synergize with chemoradiation cytotoxic effects on gynecological cancer cell lines. Additionally, we were able to show that the lack of efficacy may be attributed to an impaired mechanism of EGFR down-regulation. Nonetheless, this relative intrinsic resistance could be circumvented by the use of PI3K inhibitors that may emerge as a novel target in this tumor type.

In this study, we used a panel of gynecological cancer cell lines, with different EGFR/HER2 status, that we have previously characterized [12]. A431, a vulvar carcinoma cell line, strongly expresses EGFR, while the cervical carcinoma Caski and C33A cell lines showed moderate and low expression levels of this receptor [12]. Although bearing differences regarding EGFR expression, each one of these cell lines harbor genetic modifications that overactivate the EGFR pathway, as follows: A431 has the EGFR gene amplified (30 copies) and Caski cells harbor a PIK3CA exon 9 activating mutation (E545K), while C33A has a PTEN mutation [24-26]. These genetic lesions assure that EGFR pathway signaling is enhanced and, therefore, these cells behave as constantly activated by EGF. Nonetheless, the resulting signaling of such molecular alterations differs among these cell lines (for example, the constitutive levels of pAkt) and may differentially affect its response to PI3K/Akt pathway modulation. However, EGF-elicited signal transduction is not the only mechanism mediated by anti-EGFR MAbs, since these molecules can also induce ADCC [9] and, in primary cervical cancer cell lines obtained from cervical biopsies, ADCC induction was dependent on EGFR expression [20]. Accordingly, matuzumab effectively induced ADCC in A431 [13] and Caski cells, while no ADCC was observed in the C33A cell line, reinforcing that induction of ADCC depends on a certain level of EGFR cell surface expression.

In our previous study, we demonstrated that although A431, Caski and C33A showed different sensitivities to RxT and cisplatin, all cell lines tested showed a clearly improvement in cytotoxicity when anti-EGFR MAb cetuximab was added to chemoradiation treatments [12]. In the present study, we have shown that, unlikely cetuximab [12], matuzumab fails to induce EGFR down-regulation and chemo/radio sensitization. These preclinical findings might explain the overall unsuccessful results obtained in phase I and II studies testing matuzumab. No evidence of clinical activity was observed when matuzumab was administered as monotherapy in patients with epithelial ovarian cancer [21] and, phase II studies showed that matuzumab combined with epirubicin, cisplatin and capecitabine (ECX), or pemetrexed, does not increase response or survival of patients with advanced esophagic-gastric and NSCLC cancers, respectively [22,23]. Moreover, it was recently reported that Takeda Pharmaceutical Company Limited discontinued matuzumab development based on the negative clinical findings to date [27].

It has been recently described that derailed endocytosis is an emerging feature of cancer [28,29] and receptor down-regulation induced by anti-EGFR MAbs was described as an important mechanisms responsible for growth factor receptors inactivation and termination of EGFR cascade signaling [17,28-32]. Additionally, it has been described that EGFR accumulation on the cell membrane is responsible for cetuximab resistance in NSCLC and head and neck carcinoma cells [8,31]. Importantly, it has been reported that EGFR internalization/degradation is controlled by receptor dimerization, rather than kinase activation [33]. Moreover, based on structural studies, a model has been proposed in which matuzumab binding to EGFR prevents the conformational rearrangement required for dimerization [34]. Our data corroborate all these observations, as we described that matuzumab indeed reduced EGFR phosphorylation status, although it was not able to decrease total EGFR protein content in gynecological cancer cells, with consequent activation of downstream signaling pathways and persistent cell proliferation. Described by several authors [6-8,28-30,35-37], defective EGFR internalization/down-regulation also facilitates heterodimerization with other ErbB family members, with persistent cell signaling and survival. Accordingly, we suggested that efficient removal of EGFR from the cell surface through the induction of receptor down-regulation by MAbs is likely to reduce the oncogenic potential of the receptor. According to this hypothesis, in a previous study, we demonstrated that the use of cetuximab synergized with matuzumab through the induction of EGFR degradation and inhibition of downstream signaling pathways in A431 cells [13]. Here, we have shown that the lack of efficacy of matuzumab in monotherapy also seems to correlate to its inability to induce EGFR degradation, since proteassomal blockade in the presence of matuzumab did not induce further EGFR accumulation when compared to control. Furthermore, p-EGFR accumulation under proteassomal inhibition led to ERK/MAPK and Akt activation, corroborating the idea that degradation of EGFR is directly associated to the termination of the signaling cascade. Interestingly, cetuximab inhibited MG132-elicited p-ERK increase, but not p-Akt, suggesting that the EGFR degradation induced by this MAb is indeed necessary to its downstream effects upon PI3K/Akt pathway.

Activation of PI3K leads to plasma membrane recruitment and activation of Akt, that has been found to be a central cause of tumor-cell resistance and might have a significant role in modulating the effectiveness of ErbB-directed therapies [4,38]. Indeed, it is well known that acceleration of internalization and lysosomal targeting leads to EGFR down-regulation, which leads to a decrease in the number of activated receptors in the cell, preventing excessive signaling [28,29,32]. Importantly, activation of PI3K and protein kinase B (PKB)/Akt is thought to occur mostly at the plasma membrane compartment and is, therefore, negatively regulated by endocytosis [39].

EGFR accumulation at plasma membrane enhances the recruitment and activation of PKB/Akt proteins, and these events could be responsible for maintaining cell proliferation and survival. In the present study, the importance of the PI3K/Akt pathway in modulating the resistance to matuzumab in A431 and Caski cells was demonstrated when we combined LY294002, a specific PI3K inhibitor, which resulted in a synergistic inhibition of cell signaling, proliferation and apoptosis induction. Akt modulates cell signaling by phosphorylation of several substrates and among them is caspase-9, a protease that is activated in the apoptotic cell death pathway. Akt-phosphorylated caspase-9 is inactive and not able to trigger caspase-3 cleavage and its subsequent activation, leading to cell death blockade [5]. Here, we show that the combination of matuzumab and a PI3K inhibitor is able to induce cell death by apoptosis, suggesting that impairment of PI3K signaling releases the negative regulation exerted by this kinase upon the apoptotic machinery.

Recently, it was described that PTEN gene is mutated in C33A cells [26] and loss of PTEN protein expression induces Akt constitutive activation and proliferation of C33A cells [40]. Accordingly, in our previous study, we have shown that C33A cells expressed higher constitutive levels of p-Akt, when compared to A431 and Caski cells [12]. These findings may explain why LY294002 alone induced a markedly reduction in C33A cell survival, with no additional inhibition reached by matuzumab double treatment, since EGFR expression is almost undetectable in this cell line [12], suggesting that C33A cell survival is driven in a great extent by Akt signaling, in an EGFR-independent manner. Importantly, human papillomavirus (HPV) infection represents the most relevant risk factor for the development of cervical cancer [20]. Indeed, recently it was described that activation of the PI3-kinase/PKB/AKT pathway through the active subunit phosphatidylinositol 3-kinase catalytic alpha (PIK3CA) is essential for HPV-induced transformation *in vitro *[41]. Caski cells are HPV positive, and also harbor an activating mutation in the PIK3CA gene [25]. This cell line constitutes a pre-clinical model that represents a broad spectrum of HPV positive cervical cancer patients that, according to our results, could benefit by a combination of anti-EGFR based therapies and PI3K-Akt inhibitors.

**Figure 6:**
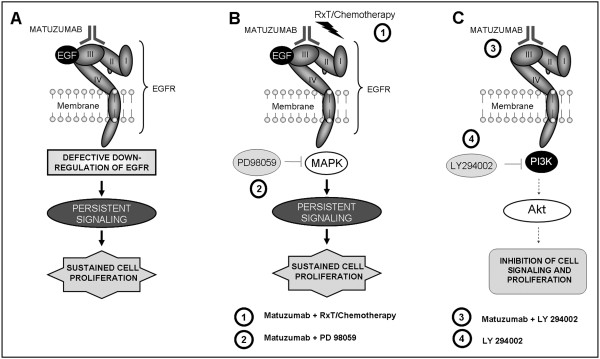
**Proposed model to explain the mechanism of ineffectiveness of matuzumab *in vitro *and how to overcome it**. (A) Matuzumab was not able to induce EGFR down-regulation, with persistent signaling and cell proliferation of gynecological cancer cells. (B) Combination of matuzumab with chemoradiation or PD98059 did not trigger benefits over single treatment. (C) Targeting PI3K with the specific inhibitor LY294002 overcomes resistance to matuzumab in EGFR positive gynecological cancer cell lines.

Based on these findings, we proposed a model (Figure 6) that explains one possible mechanism of ineffectiveness of matuzumab and how to overcome it. Matuzumab, differently from cetuximab, was not able to induce EGFR down-regulation, with persistent signaling and gynecological cancer cell proliferation (Figure 6A). Although the combination of matuzumab with chemoradiation or a MAPK pathway inhibitor did not trigger benefits over single treatments (Figure 6B), we observed that targeting PI3K, in combination with matuzumab, markedly reduced A431 and Caski cell survival, highlighting the importance of PI3K/Akt pathway (Figure 6C).

The present report is the first one to bring out preclinical studies showing matuzumab resistance *in vitro *in gynecological cancer cell lines and highlights that impaired EGFR down-regulation might be the possible biological mechanism responsible for its inefficacy. Even though the majority of gynecological cancers express EGFR [42], these tumors are not solely dependent upon EGFR activity. This is likely due to the presence of preexisting or treatment-induced compensatory signaling pathways. Since EGFR signaling involves intracellular interactions with other oncogenic pathways, it is plausible that cotargeting of EGFR in rational combination with specific inhibitors of these pathways may achieve a more potent antitumour effect and help to overcome the development of resistance, an emerging clinical issue often responsible for the failure of most modern antitumour approaches. These results indicate that Akt pathway and EGFR may not be completely responsible, but cooperate in the resistance of gynecological cancer cells to matuzumab and suggest a rationale for the design of clinical strategies directed to patients displaying a resistant profile to anti-EGFR therapies. Our results, along with the knowledge that different signal transduction pathways controls tumor growth and are connected to resistance, suggest that future therapeutic approaches are likely to involve the combination of different antineoplastic targeted agents.

## Competing interests

The authors declare that they have no competing interests.

## Authors' contributions

DM carried out MTT and clonogenic assays, Western blotting, participated in the design of experiments, performed the statistical analysis and drafted the manuscript. VA carried out MTT, clonogenic, ADCC and cell death assays, Western blotting and participated in the design of experiments. JM carried out MTT and clonogenic assays. MC carried out cell death assays. IN carried out flow cytometry analysis. DB developed the settings for the irradiation experiments. CS participated in the discussion and interpretation of the study and manuscript preparation. CG conceived the study and participated in its design, coordination and manuscript preparation. All authors read and approved the final manuscript.

## Abbreviation List

ADCC: antibody-dependent cellular cytotoxicity; CA: clonogenic assay; CC: cervical cancer; ECL: enhanced chemiluminescence; EGF: epidermal growth factor; EGFR: epidermal growth factor receptor; ERK 1/2: extracellular signal-regulated kinase; E/T: effector/target ratios; MAbs: monoclonal antibodies; MAPK: mitogen-activated protein kinase; MTT: 3-(4,5-dimethythiazol-2-yl)-2,5-diphenyltetrazolium bromide; PBMC: peripheral blood mononuclear cells; PI: propidium iodide; PI3K: phosphatidylinositol 3-kinase; TKI: tyrosine kinase inhibitor; SF: surviving fraction; WB: Western blotting.
